# Association between estrogen receptor alpha 36 expression and the risk of deep infiltrating endometriosis

**DOI:** 10.3389/fendo.2026.1752870

**Published:** 2026-03-11

**Authors:** Ying Zhang, Tiantian Yu, Chunbo Zhao, Ruihong Xue

**Affiliations:** 1 The International Peace Maternity and Child Health Hospital, School of Medicine, Shanghai Jiao Tong University, Shanghai, China; 2 Shanghai Key Laboratory of Embryo Original Diseases, Shanghai, China; 3 Institute of Birth Defects and Rare Diseases, School of Medicine, Shanghai Jiao Tong University, Shanghai, China; 4 Department of Obstrics and Gynecology, The First People’s Hospital of Jiande, Hangzhou, China

**Keywords:** deep infiltrating endometriosis (DIE), diagnostic biomarker, endometriosis, ERα36, estrogen, gynecologic disorders, pain

## Abstract

**Background:**

Deep infiltrating endometriosis (DIE) is a severe subtype of endometriosis, often associated with dysmenorrhea and significant diagnostic challenges. Conventional biomarkers, such as cancer antigen 125 (CA125), lack sufficient specificity for accurate DIE diagnosis. Estrogen receptor α36 (ERα36) is a novel isoform of the estrogen receptor, distinct from the classic estrogen receptor α66 (ERα66), and has been implicated in the pathogenesis of several gynecologic disorders, including breast and endometrial cancer. However, the expression pattern of *ERα36* in DIE tissues and its potential role as a diagnostic biomarker for DIE have not yet been investigated. This study aimed to examine the expression of *ERα36* in DIE tissues, explore its association with disease presence, and evaluate its potential as a diagnostic biomarker and a candidate for future therapeutic targeting.

**Methods:**

A case-control study was conducted between 2014 and 2016, enrolling 80 DIE patients as the case group and 132 healthy women as the control group. The expression of ERα36 was evaluated at both the mRNA and protein levels: reverse transcription-polymerase chain reaction was used for mRNA expression and immunohistochemistry was performed for protein detection. Statistical analyses were performed using SPSS version 26.0, and receiver operating characteristic curve analysis was employed to assess the diagnostic performance of ERα36 for DIE.

**Results:**

No significant differences were observed between control and endometriosis groups in demographic or reproductive characteristics (all *P*>0.05). ERα36 mRNA was detected in 71.2% of endometriosis tissues, compared with 36.4% of controls (*P* < 0.01). Among 80 patients with endometriosis, ERα36 expression was observed in 57 cases and absent in 23. ERα36-positive patients exhibited younger age, higher rFAS (revised American Fertility Society) stage, more severe and longer-lasting dysmenorrhea, increased absenteeism during menstruation, greater adnexal adhesions, and a higher prevalence of DIE (all *P* < 0.05). Protein gene product 9.5 (PGP9.5) was present in eutopic endometrium, with significantly higher density in DIE lesions compared with ovarian and superficial peritoneal endometriosis (*P* < 0.01). Notably, ERα36-positive patients showed a higher proportion of strong *PGP9.5* expression than ERα36-negative patients (*P* < 0.05). These findings indicate that *ERα36* expression is associated with more severe disease phenotypes and increased neural marker expression in endometriosis, particularly in DIE.

**Conclusion:**

ERα36 is highly expressed in DIE tissues and exhibits good diagnostic performance with high sensitivity and specificity. These findings suggest that ERα36 may serve as a novel tissue-level biomarker for DIE, representing a potential target for future therapeutic strategies.

## 1 Introduction

Endometriosis is an estrogen-dependent condition characterized by the presence of endometrial glands and stroma outside the uterus and is clinically associated with pain and infertility (1). About 6%–10% of women of reproductive age are affected. Although endometriosis is histopathologically benign, it exhibits tumor-like behavior, including growth, infiltration, and adhesion to surrounding tissues (2). Based on histological features, endometriosis is classified into superficial peritoneal endometriosis (SUP), ovarian endometrioma (OMA) and deep infiltrating endometriosis (DIE). DIE is defined as endometriotic lesions penetrating more than 5 mm beneath the peritoneal surface and is characterized by glandular activity that is often synchronized with the menstrual cycle (3). DIE commonly involves the uterosacral ligaments, posterior vaginal wall, bowel, bladder, and ureter (4). Patients with untreated DIE usually present with high preoperative pain scores, because these lesions are associated with pelvic pain syndromes, including dysmenorrhea, deep dyspareunia, noncyclic chronic pelvic pain, and gastrointestinal and lower urinary tract symptoms (5). Moreover, the need for repeated medical treatment and invasive diagnostic procedures often results in prolonged or recurrent absence from work, imposing substantial physical, psychological, and economic burdens, particularly in patients with DIE. Consequently, many patients experience significant mental distress, and some may develop depression (4). Despite its clinical importance, the underlying mechanisms driving DIE remain poorly understood.

Estrogen receptor alpha plays a cardinal role in mediating the transcriptional effects of estrogen and is also involved in regulating proliferation, survival, apoptosis, and differentiation across diverse cell types. Estrogen receptor alpha 36 (ERα36) is a recently identified variant of the wild type of ERa66, with a molecular weight of 36 kDa, first discovered and cloned by Wang et al. in 2005 (6). Compared with ERa66, ERα36 retains the DNA-binding/dimerization and partial ligand-binding domains, but lacks the two transcriptional activation domains, AF-1 and AF-2. Instead, ERα36 has a unique C-terminal 27 amino acid domain that replaces the final 138 amino acids encoded by exons 7 and 8 of ERa66 (6). Owing to these structural features, ERα36 is thought to possess a broad ligand-binding spectrum and may contribute to the diverse biological effects observed in estrogen-dependent diseases.

Estrogen receptors (ERs) are known to participate in various processes in steroid-responsive cancers (7), making ERα36 a promising biomarker for estrogen-dependent cancer. In breast cancer, ERα36 expression is positively associated with tumor size, lymph metastasis, disease severity, and short survival of patients (7). In addition, ERα36 has been shown to localize to the plasma membrane and cytoplasm in endometrial cancer cells, and its expression correlates with the clinical stage, pathological grade, and poor disease-free survival in endometrial tumors (8). However, the role and mechanistic contribution of estrogen receptor α36 (ERα36) in endometriosis remain largely unexplored.

Here we examined ERα36 mRNA expression in eutopic endometrium of patients with and without endometriosis, aiming to investigate the association between ERα36 expression and the clinicopathological characteristics of endometriosis.

## 2 Methods

### 2.1 Study population

The endometrium samples were collected from women of reproductive age from 2014 to 2016. All participants were Chinese women of Han ethnicity who underwent surgery at the Department of Gynecology and Obstetrics, International Peace Maternity & Child Health Hospital, School of Medicine, Shanghai Jiao Tong University. The approval was obtained from the institutional ethics committee and written informed consent was secured from all patients prior to participation. None of the patients had received hormonal therapy within the 3 months preceding surgery.

The patient’s menstrual cycle phase was determined based on gynecological anamnesis, endometrium examination, and histological analysis performed immediately prior to surgery. Endometrial biopsies were obtained during the mid-proliferative or secretory phases. All patients underwent laparoscopic peritoneal biopsies, and the diagnosis of endometriosis was established by identification of peritoneal endometriotic lesions and subsequently confirmed by histological evaluation by an experienced pathologist. The severity of pelvic pain symptoms prior to surgery was assessed using a 10-point visual analogue scale (VAS).

Patients diagnosed with endometriosis by laparoscopy or laparotomy and confirmed by histopathological examination were enrolled as the case group. During the same period, patients who underwent surgery for uterine fibroids or benign ovarian tumors (including teratoma, simple ovarian cysts, or parovarian cysts), or for cervical intraepithelial neoplasia, and in whom no endometriosis was detected by intraoperative exploration or pathological examination, were recruited as the control group. All patients in both groups had relatively regular menstrual cycles (28–32 days), normal menstrual flow, and were premenopausal women. Exclusion criteria: Within six months prior to surgery, patients with concomitant medical or surgical diseases, intrauterine device use, hormonal therapy, a history of pregnancy or lactation, chronic pelvic inflammatory disease, or malignant tumors were excluded from the study.

### 2.2 RNA isolation

Total RNA was extracted from frozen endometrial tissue samples using the Purelink RNA Mini Kit (ThermoFisher, Foster City, CA). RNA quantity and purity were assessed by spectrophotometric analysis using a Nanodrop 2000 (ThermoFisher, Foster City, CA) at absorbance wavelengths of 260 nm and 280 nm, and the 260/280 and 260/230 ratios were calculated. Next, reverse transcription was performed to synthesize cDNA from total RNA using the Superscript IV First-Strand Synthesis System (ThermoFisher Scientific, Waltham, MA) according to the manufacturer’s instructions.

### 2.3 Reverse Transcription Polymerase Chain Reaction, cloning, and DNA sequencing

RT-PCR was performed in a 20 µL reaction mixture containing cDNA equivalent to 1 µg of total RNA, 1×PCR buffer, 0.2 mM dNTP mixture, 0.2 µM of forward and reverse primers, and 0.25U of Q5 Hot Start High-Fidelity DNA polymerase (NEB, UK). The PCR cycling conditions consisted of an initial denaturation at 95 °C for 2 min, followed by cycles of denaturation at 95 °C for 5 s, annealing at 60 °C for 30 s, and extension at 72 °C for 60 s, with a final elongation step at 72 °C for 2 min. The oligonucleotide primer sequences for RT-PCR are provided in the **Supplementary Information**. The PCR products were resolved on 2% agarose gels, excised, purified, and cloned into the pCR2.1-TOPO vector for DNA sequence analysis (Majorbio Company, Shanghai, China).

### 2.4 Semi-quantitative PCR

Semi-quantitative PCR amplification was performed using OneTaq^®^ DNA Polymerase (New England Biolabs; M0480) in a 25 μL reaction mixture. The reaction contained 1× OneTaq Standard Reaction Buffer, 0.2 mM dNTPs, 0.5 μM forward primer, 0.5 μM reverse primer, 1 U OneTaq DNA Polymerase, and 50–100 ng of template DNA. The thermal cycling conditions were as follows: initial denaturation at 98 °C for 30s; 38 cycles of denaturation at 98 °C for 5 s, annealing at 65 °C for 20s, and extension at 72 °C for 2 min. Amplification products were analyzed by 1.5% agarose gel electrophoresis, stained with ethidium bromide, and visualized under UV illumination. Conventional β-actin primer sequences (5′-3′) for common species: Forward primer GCTSGTCGTCGACAACGGCTC, Reverse primer CAAACATGATCTGGGTCATCTTYTC, Gel imaging and grayscale quantification were performed immediately after electrophoresis. The gel was photographed using a gel documentation system (302 nm UV, consistent exposure) for comparability. Target band grayscale intensity was quantified with ImageJ software (Version 1.8.0, NIH, USA): after grayscale conversion and background subtraction, integrated density values (IDV) were measured, and PCR product relative abundance was determined by normalized band intensity (to internal reference). All experiments were performed in triplicate (≥3 independent repeats) for reproducibility, with mean ± SD of normalized IDVs used for statistical analysis. A no-template control (NTC) was included to exclude cross-contamination, and only samples with clear target bands (no obvious nonspecific bands) were quantified.

### 2.5 Immunohistochemistry

The tissues were fixed in 4% paraformaldehyde for 24 h, embedded in paraffin, and sectioned at 4 μm thickness and mounted onto glass slides. After deparaffinization, dehydration, rehydration, and antigen retrieval, the endogenous peroxidase activity was deactivated with H_2_O_2_ and the nonspecific binding was blocked by bovine serum albumin. Sections were incubated with the primary antibody (1:1000) overnight at 4 °C, followed by incubation with the appropriate secondary antibody. After visualization with 3,3′-diaminobenzidine and counterstaining with hematoxylin staining, the slides were mounted using Permount™ Mounting Medium. Six random fields per section were captured using a Leica DM2000 microscope. The integrated optical density was quantified using Image-Pro Plus software version 6.0. A positive signal for PGP9.5 staining was defined as the presence of yellow-brown granules in the cytoplasm. Scoring was performed by integrating staining intensity and the proportion of positive cells. The area with the strongest expression was selected and scored according to staining intensity as follows: no staining or staining similar to background = 0 points; weak staining, slightly higher than background = 1 point; moderate staining, clearly higher than background = 2 points; strong staining, dark brown = 3 points. The proportion of positive cells was scored as follows: <25% = 0 points; 25%–50% = 1 point; 51%–75% = 2 points; >75% = 3 points. The two scores were summed and classified into four grades: 0–1 points (−), 2 points (+), 3–4 points (++), and 5–6 points (+++). Cases graded (−) were considered negative, whereas those graded (+) to (+++) were considered positive.

### 2.6 Statistical analysis

Statistical analyses were performed using SPSS (v16.0 for Windows, SPSS Inc., Chicago, IL). Descriptive data are presented as mean ± standard deviation, while categorical variables are expressed as counts and percentages. Normally distributed data were compared between groups using Student’s *t*-test, whereas non-normally distributed variables were analyzed using the Mann–Whitney *U* test. Multivariable logistic regression was performed to determine whether *ERα36* expression is independently associated with DIE. Correlations between the number of positively stained domains and symptom severity were assessed using Spearman’s rank correlation coefficient. Image analysis was performed using Image-Pro Plus software version 6.0. The comparison of immunohistochemical PGP9.5 expression levels between the normal endometrium group and the endometrial carcinoma group was performed using the nonparametric Wilcoxon rank-sum test. A Mann–Whitney U test was also used to assess differences, and statistical significance was defined as *P* < 0.05.

## 3 Results

### 3.1 Clinical characteristics of patients with and without endometriosis

As shown in **Table 1**, no significant correlation between control and endometriosis groups with respect to age, body mass index (BMI), education level, occupation, income, place of residence, smoking status, alcohol consumption, exercise habit, parity, or history of abortion or curettage (all *P*>0.05).

**Table 1:** Clinical characteristics of patients with or without endometriosis.

Clinical characteristic	Control (n = 132)	Endometriosis (n = 80)	*P-*value
Age (y)	40.2 ± 7.25	39.75 ± 8.42	0.68
Body mass index (kg/m^2^)	22.51 ± 3.92	21.49 ± 2.72	0.04
Age at menarche (y)	14.21 ± 2.74	13.95 ± 3.10	0.52
Highest education (n, %)			
Middle school or less	63 (47.7)	29 (36.3)	0.06
High school or University	51 (38.6)	36 (45)	
Postgraduates or above	18 (13.6)	15 (18.8)	
Occupation			
Mental status	57 (43.2)	34 (42.5)	0.52
Physical status	75 (56.8)	46 (57.5)	
Income RMB/m (n)			
<5000	57 (43.2)	33 (41.3)	0.32
5000–20,000	63 (47.7)	37 (46.3)	
>20000	12 (9.1)	10 (12.5)	
Residence (n)			
Urban	73 (55.3)	36 (45)	0.15
Rural	59 (44.7)	44 (55)	
Smoker (n)			
Never	101 (76.5)	58 (72.5)	0.51
Former or Current	31 (23.5)	22 (27.5)	
Alcohol use (n)			
Less than monthly	70 (70.5)	43 (53.8)	0.92
More than monthly	62 (29.5)	37 (46.3)	
Regular exercise (n)			
No	60 (45.5)	37 (46.3)	0.9
Yes	72 (54.5)	43 (53.8)	
Parity (n)			
Nulliparous	51 (38.6)	34 (42.5)	0.58
Parous	81 (61.4)	46 (57.5)	
Abortion or Curettage (n)			
No	60 (45.5)	35 (43.8)	0.81
Yes	72 (54.5)	45 (56.3)	

*p-values were calculated using Student’s t-test or Chi-square test.

### 3.2 ERα36 expression profiles in endometrial tissues

**Figure 1:**
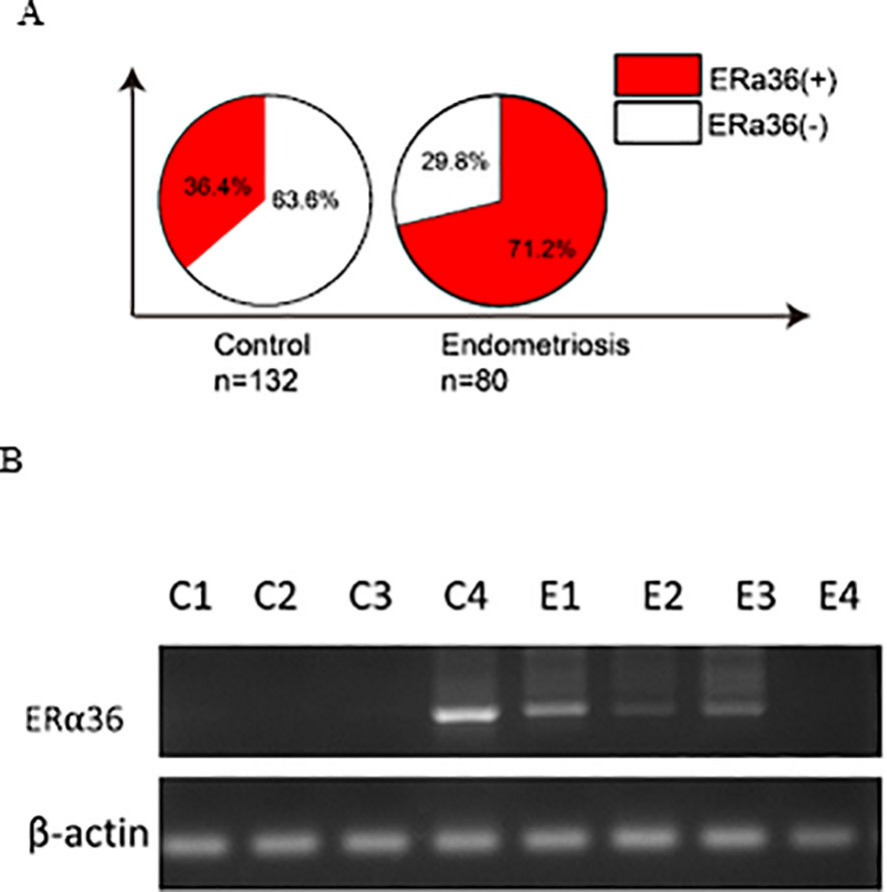
Comparison of *ERα36 expression between* endometriosis vs. non- endometriosis tissues. **(A)** Percentage distribution of *ERa36* expression in control (n=132) and endometriosis (n=80) groups. ERα36, estrogen receptor α36. **(B)** Representative PCR image showing *ERα36* expression in endometrium with and without endometriosis. ERα36: estrogen receptor α36.

As shown in **Figure 1A**, ERα36 mRNA was detected in 36.4% non-endometriosis tissues and in 71.2% endometriosis tissues. ERα36 expression was significantly higher in endometriosis tissues compared with non-endometriosis controls (*P* < 0.01) (**Supplementary Figure 1**; **Table 1**). **Figure 1B** shows representative PCR images of gel electrophoresis.

### 3.3 Association of ERα36 expression with clinicopathological characteristics in patients with endometriosis

The clinicopathological characteristics of endometriosis patients stratified by expression of ERα36 are summarized in **Table 2**. Among the 80 endometriosis patients, ERα36 mRNA was detected in 57 patients and was undetectable in 23 patients. Significant differences were observed between ERα36-positive and ERα36-negative patients with respect to age, rFAS stage, distribution of worst endometriotic lesion, adnexal adhesions, Douglas obliteration condition, dysmenorrhea VAS score, dysmenorrhea duration, and absenteeism from school during menstruation (*P* < 0.05). Notably, endometriosis patients with ERα36 expression exhibited a more severe disease phenotype, characterized by younger age, higher rFAS stage, more severe dysmenorrhea, and longer dysmenorrhea duration. This may be partly attributed to the higher proportion of patients with DIE in the ERα36 expression group (*P* < 0.05). In contrast, no significant differences were observed between the two groups in terms of BMI, age at menarche, family history of endometriosis, coexisting OMA, or SUP, cancer antigen 125 (CA125) or CA199 levels, menstruation blood amount, menstruation period, menstruation cycle, parity, history of abortion or curettage, vaginal delivery, cesarean delivery, deep dyspareunia, noncyclic chronic pelvic pain, gastrointestinal symptoms, lower urinary tract, and history of infertility.

**Table 2:** Pathological characteristics of endometriosis patients with ERα36+ or ERα36- expression.

	ERα36 (+) (n=57)	ERα36 (-) (n=23)	*P-*value
Age at surgery (y)	35.7 ± 5.8	36.4 ± 7.2	0.65
BMI (kg/m^2^)	21.63 ± 0.96	21.92 ± 1.20	0.26
Age at menarche (y)	13.89 ± 2.23	13.77 ± 3.28	0.85
rFAS stage (n, %)			
I–II	12 (21.2)	8 (21.7)	0.10
III–IV	45 (78.)	15 (65.2)	
Family history of endometriosis (n, %)			
NO	26 (45.6)	9 (15.8)	0.60
YES	31 (54.4)	14 (60.9)	
Distribution according to the endometriotic lesion (n, %)			
Combined with DIE			
NO	38 (33.3)	21 (73.9)	0.023
YES	19 (66.7)	2 ((26.1)	
Combined with OMA			
NO	26 (45.6)	5 (21.7)	0.70
Single	16 (28.1)	10 (43.5)	
Bilateral	15 (26.3)	8 (34.8)	
Combined with SUP			
NO	21 (36.8)	11 (47.8)	0.36
YES	36 (63.2)	12 (52.1)	
Douglas obliteration (n, %)			
Absent	9 (15.8)	9 (39.1)	0.01
Partial	22 (38.6)	9 (39.1)	
Complete	26 (45.6)	5 (21.7)	
Adnexal adhesions (n, %)			
NO	30 (52.6)	14 (60.9)	0.503
YES	27 (47.4)	9 (39.1)	
CA125 (n, %)^a^			
Normal	21 (36.8)	11 (45.4)	0.45
Increased	32 (56.1)	9 (39.1)	
No examination	4 (7)	3 (13)	
CA199 (n, %)^b^			
Normal	28 (49.1)	13 (56.5)	0.237
Increased	16 (28.1)	7 (30.4)	
No examination	13 (22.8)	3 (13.0)	
Menstruation blood amount (n, %)			
Normal	37 (64.9)	16 (69.6)	0.45
More than usual	20 (35.1)	7 (30.4)	
Menstruation period (n, %)			
Regular (3–7 days)	43 (75.4)	15 (65.2)	0.35
Irregular (<3 or >7days)	14 (24.6)	8 (34.8)	
Menstruation cycle (n, %)			
Regular (28–35 days)	40 (70.2)	14 (60.9)	0.42
Irregular (<28 or >35 days)	17 (29.8)	9 (39.1)	
Parity (n, %)			
Nulliparous	11 (19.3)	3 (13.0)	0.51
Parous	46 (80.7)	20 (87.0)	
Abortion or curettage (n, %)			
Never	15 (26.3)	5 (21.7)	0.35
1–3 times	30 (52.6)	12 (52.2)	
≥3 times	12 (21.1)	6 (26.1)	
Vaginal delivery (n, %)			
NO	21 (45.7)	11 (55.0)	0.49
YES	25 (54.3)	9 (45.0)	
Cesarean delivery (n, %)			
NO	25 (54.3)	9 (45.0)	0.49
YES	21 (15.7)	11 (55.0)	
Dysmenorrhea (VAS score) (n, %)			
0–3	8 (14.0)	6 (26.1)	0.015
4–6	22 (38.6)	13 (56.5)	
7–10	27 (47.4)	4 (17.4)	
Dysmenorrhea duration (n, %)			
Less than 5 years	13 (22.8)	12 (52.2)	0.008
5–10 years	26 (45.6)	8 (34.8)	
More than 10 years	18 (31.6)	3 (13.0)	
Absenteeism from school during menstruation (n, %)			
NO	20 (35.1)	16 (69.6)	0.005
YES	37 (64.9)	7 (30.4)	
Before surgery do patients feel			
Deep dyspareunia (n, %)^c^			
Never	9 (15.8)	10 (43.5)	0.008
Sometimes	27 (47.4)	9 (39.1)	
Usual	21 (36.8)	4 (17.4)	
Noncyclic chronic pelvic pain (n, %)			
Never	4 (17.0)	15 (65.2)	0.00
Sometimes	31 (31.0)	9 (39.1)	
Usual	22 (38.6)	4 (17.4)	
Gastrointestinal symptoms (n, %)			
Never	11 (19.3)	15 (65.2)	0.00
Sometimes	27 (47.4)	5 (21.7)	
Usual	19 (33.3)	3(13.0)	
Lower urinary tract symptoms (n, %)			
Never	14 (24.6)	13 (56.5)	0.055
Sometimes	27 (47.4)	4 (17.4)	
Usual	16 (28.1)	6 (26.1)	

^a, b^Patients did not undergo CA125 or CA199 testing; c, patients were not sexually active; d, patients were unmarried and their fertility status was unknown.

*p-value were calculated using Student’s t-test or Chi-square test.

### 3.4 PGP9.5 expression in the endometrium

**Figure 2:**
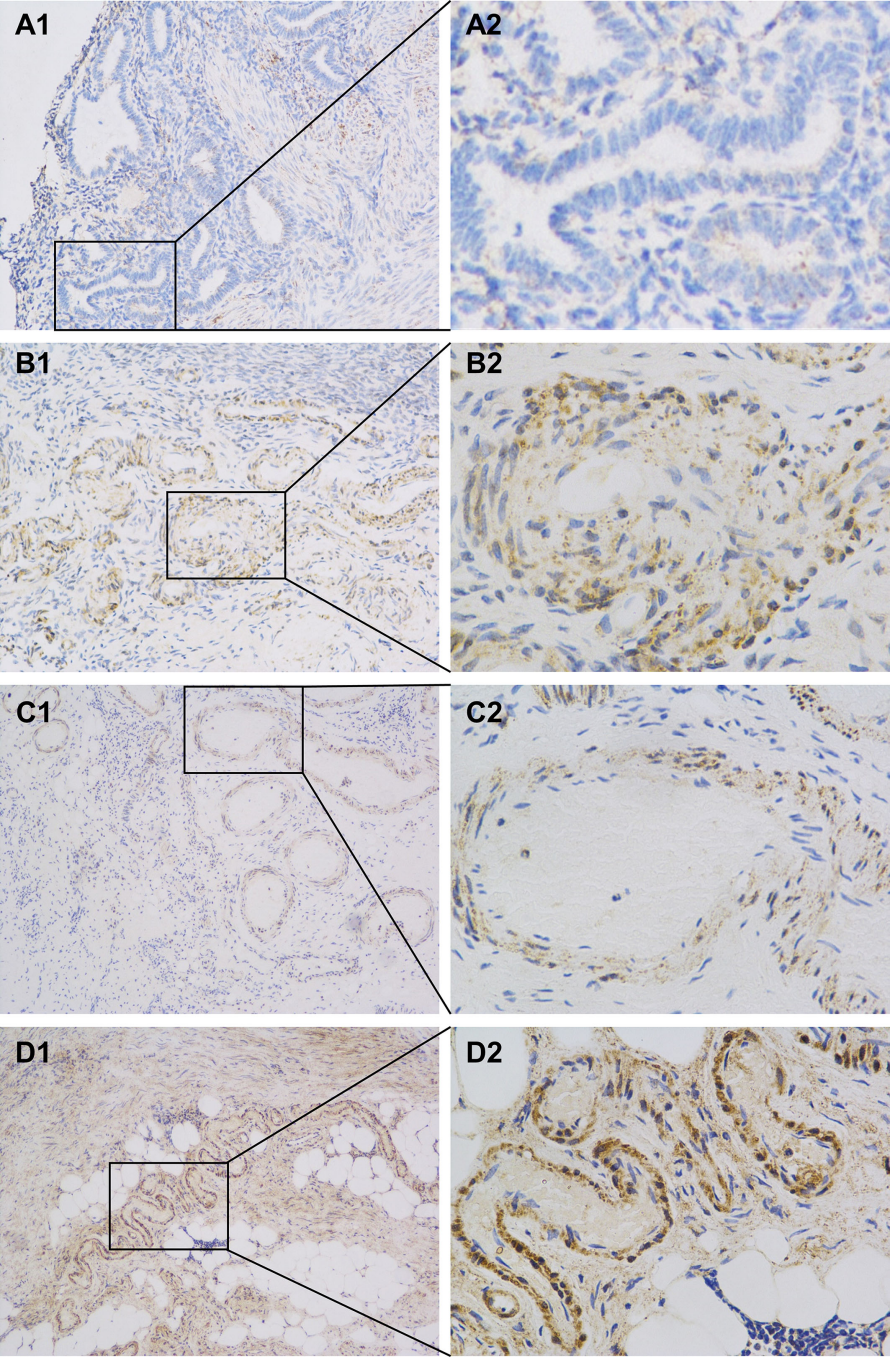
Expression of PGP9.5 in eutopic and ectopic endometrium measured by Immunohistochemistry. **(A1,2)** Expression of PGP9.5 in the functional layer of eutopic endometrium (SP, 100×, 400×). **(B1,2)** Expression of PGP9.5 in ovarian endometrioma. (SP, 100×, 400×). **(C1,2)** Expression of PGP9.5 in superficial peritoneal endometriosis (SP, 100×, 400×). **(D1,2)** Expression of PGPG9.5 in deep infiltrating endometriosis of uterosacral ligaments. PGP9.5, protein gene product 9.5; SP, superficial peritoneal.

**Figure 3:**
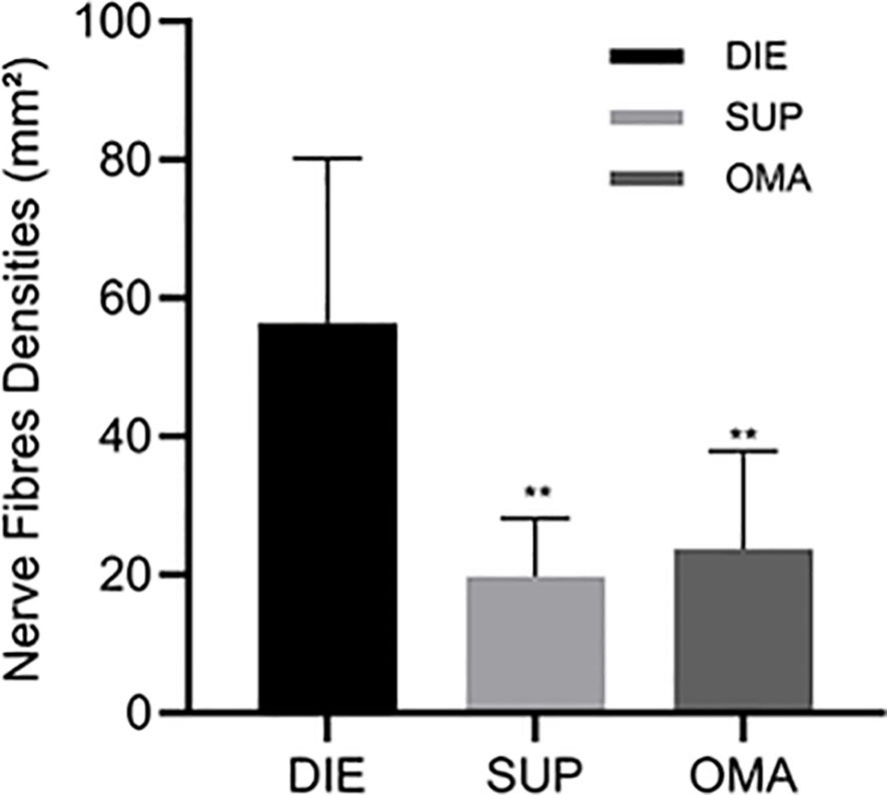
Nerve fiber density in endometriotic lesions. Quantification of nerve fiber densities (mm²) in deep infiltrating endometriosis (DIE) (n=18), superficial peritoneal lesions (SUP) (n=31), and ovarian endometrioma (OMA) (n=24). DIE lesions exhibited the highest nerve fiber density, whereas SUP and OMA showed significantly lower densities. Bars represent mean ± SD.; **P < 0.01 vs. DIE.

Protein gene product 9.5 (PGP9.5), a neuroectoderm-derived, tissue-specific marker, was used to label nerve fibers in this study. Nerve fibers were observed in the eutopic endometrium of patients with endometriosis (**Figures 2A1, A2**). Quantitative analysis of nerve fiber distribution among different types of endometriotic lesions was performed using Image-Pro Plus software. Nerve fiber density in DIE lesions (**Figures 2D1, D2**) was significantly higher than that observed in ovarian endometriosis (**Figures 2B1, B2**) and superficial abdominal endometriosis (**Figures 2C1, C2**) (*P* < 0.01) (**Figure 3**), while no significant difference was observed in nerve fiber density between ovarian endometriosis and superficial abdominal endometriosis.

### 3.5 Association between ERα36 and PGP9.5 expression

Analysis of the distribution differences in PGP9.5 expression intensity according to Erα36 status showed a statistically significant difference in overall distribution between the Erα36(+) group (n = 57) and the Erα36(−) group (n = 23) (*P* < 0.05) (**Table 3**). The Erα36(+) group was predominantly characterized by high PGP9.5 expression, with PGP9.5+++ accounting for 33.3% and PGP9.5++ for 36.8%, representing a combined proportion of 70.1%. In contrast, the Erα36(−) group was mainly characterized by low-to-moderate expression, with PGP9.5+ accounting for 39.1%, while cases with high PGP9.5 expression (+++/++) comprised only 43.4% (**Table 3**).

**Table 3:** Correlation between *ERα36* and *PGP9.5* expression in endometriosis endometrium.

PGP9.5 density	ERα (+) n=57(n, %)	ERα (-) n=23 (n, %)	*P-*value
+++	19 (33.3)	5 (21.7)	<0.05
++	21 (36.8)	5 (21.7)	
+	10 (17.5)	9 (39.1)	
–	7 (12.2)	4 (17.4)	

*P* < 0.05 indicates a statistically significant difference in the overall distribution between the two groups as determined by the χ² test.

## 4 Discussion

DIE represents the severe and clinically challenging form of endometriosis and is frequently associated with profound pelvic pain, organ dysfunction, and reduced quality of life. Despite its significant clinical impact, the pathogenesis of DIE remains poorly understood, and the marked heterogeneity of endometriotic lesions, together with the limited accessibility of deeply infiltrating tissues, has impeded the identification of reliable biomarkers that reflect disease aggressiveness and pain severity. In this context, our study demonstrates that ERα36, which lacks the classical transcriptional activation domains AF-1 and AF-2 but retains the DNA-binding/dimerization domain and part of the ligand-binding domain, is highly expressed in endometriosis and is associated with an increased risk of DIE and more severe clinical manifestations. ERα36 expression has been identified in ER-negative breast cancer cell lines (9). The development and progression of ER-negative breast cancer are considered to be independent of classical estrogen signaling (9). Clinically, ER-negative breast cancers are non-responsive or less responsive to anti-estrogen therapies and are associated with a higher risk of recurrence and metastasis, compared with ER-positive breast cancers. Notably, ERa-knockdown remarkably reduces the migratory and invasive capacities of ER-negative breast cancer cells, suggesting that Era-related pathways may represent potential therapeutic targets for limiting metastasis in this aggressive subtype (10). In breast cancer cells, ERα36 expression is predominantly localized to the cytoplasm and plasma membrane, with minimal or absent nuclear localization, consistent with its proposed role in non-genomic estrogen signaling (10). These prior findings provide a biological framework that may be relevant to endometriosis; however, whether similar mechanisms operate in DIE remains unknown.

It remains controversial whether and through which mechanisms plasma-membrane-localized ER triggers estrogen signaling. ERα36 is predominantly localized to the plasma membrane and has been shown to stimulate cell growth in response to both estrogens and antiestrogens through activation of the MAPK/ERK signaling pathway (11). These findings suggest that both estrogens and antiestrogens can boost cell proliferation via membrane-associated EERα36. In breast cancer cells, ERα36 is generally absent from the nucleus and is primarily detected at the plasma membrane, regardless of ER-positive or ER-negative status (12). Through activation of the MAPK/ERK and PI3K/AKT signaling pathways, ERα36 modulates estrogen- and anti-estrogen–dependent signaling and promotes cellular proliferation (11). Consistent with a non-genomic mode of action, transient co-transfection using a luciferase-expressing reporter containing two estrogen response elements (EREs) upstream of the thymidine kinase promoter demonstrated that ERα36 lacks intrinsic transcriptional regulatory activity, both in the presence and absence of ER-β (12). Although these mechanisms have been characterized in cancer models, their relevance to endometriosis remains speculative and requires direct experimental validation.

ERα36 strongly suppressed the transactivation mediated by the AF-1 and AF-2 domains of both ERa66 and ER-β, indicating that it lacks intrinsic transcription activity (12). Acting as a natural domain-negative regulator, ERα36 modulates genomic estrogen signaling by interfering with the AF-1 and AF-2 domains of estrogen receptor α66 (ERα66) and estrogen receptor β (Erβ) (9). Through its intact dimerization domains, ERα36 can form heterodimers with ERa66 or Erβ, thereby suppressing their transcription activities (9). In addition, its preserved DNA-binding domain allows ERα36 to occupy the same ERE sequences as ERα66 and ERβ and may retain ERα66 in the cytoplasm, preventing its genomic signaling in the nucleus (12). Nevertheless, the precise mechanisms by which ERα36 inhibits genomic estrogen signaling remain to be fully elucidated.

DIE is a special type of endometriosis characterized by lesions that penetrate more than 5 mm beneath the peritoneal surface. DIE can involve critical structures such as the uterosacral ligament, bowel, and ureters, often resulting in severe pains during menstruation, sexual intercourse, or defecation (13). Management of DIE is designed according to the patient’s clinical condition, with the primary goal of alleviating pain and improving fertility (14). Preoperative counseling typically includes a thorough discussion of fertility intentions, potential side effects of medical therapy, surgical risks, and patient preferences (14). Pharmacological treatments for DIE have generally shown limited or transient efficacy in relieving pain, whereas, surgical intervention is often more appropriate and effective (13). Nevertheless, surgical outcomes can vary considerably and are highly dependent on the experience and skill of the treating gynecologists.

Lesions that infiltrate more than 5 mm beneath the peritoneal surface represent a distinct and more aggressive form of endometriosis. These deep infiltrating lesions often cause pelvic distortion and severe pain due to their increased activity and invasiveness (15). In recent years, the pathogenesis of deep endometriosis has been the subject of debate, with growing evidence suggesting that DIE may represent a disease entity distinct from peritoneal or ovarian endometriosis (16). Some researchers have hypothesized that these lesions do not arise from retrograde menstruation and implantation of endometrial tissue but rather develop from metaplasia of Müllerian remnants located in the rectovaginal septum, consistent with the *in situ* metaplasia theory.

DIE-induced neural invasion has been shown to be closely associated with dysmenorrhea. A likely explanation for this association is that lesions penetrating the wall of adjacent organs, such as the vagina or rectum, exert a stronger impact on nerve fibers compared with non-infiltrating lesions (17).

Increasing evidence suggests that endometriosis-associated pain is closely related to nerve growth and vascular growth (17). Menstrual blood can promote the release of inflammatory factors, which in turn stimulate peripheral nerves and contribute to menstrual abdominal pain (17). The nerve fiber distribution in DIE lesions has been a particular focus of research, and several studies have reported a positive correlation between the number and density of nerve fibers and the severity of pain (18). Consistent with these findings, our present study found that, compared with SUP and OMA, DIE lesions in the uterosacral ligament exhibited a higher density of nerve fibers. Our findings reveal a clear association between nerve density and lesion severity, supporting the need for future mechanistic studies to define how neural remodeling contributes to disease progression.

PGP9.5 is an evolutionarily conserved protein and a specific marker of the neuroendocrine system derived from neuroectodermal cells. It is a component of the ubiquitin-proteasome system and is widely expressed throughout all stages of neuronal differentiation (19). PGP9.5 participates in the metabolism of apoptotic proteins in cells and plays a vital role in cell survival. Apart from the involvement of various nerve related diseases and the recurrence and prognosis of malignant tumors, PGP9.5 also regulates nervous system regeneration and tumor cell invasion (20). The high expression of PGP9.5 may promote the growth, invasion and infiltration of endometrium. Previous studies have shown a positive correlation between PGP9.5 expression and dysmenorrhea, with higher levels of PGP9.5 associated with more severe pain. These findings are consistent with our study, in which patients with DIE exhibited both severe dysmenorrhea and increased PGP9.5 expression. While this association supports a link between neural markers and pain severity, a mechanistic relationship between ERα36 and neural growth needs to be investigated.

In summary, ERα36 is closely related to the pathogenesis of endometriosis, especially DIE. The increased expression of ERα36 in DIE lesions, together with the elevated expression of PGP9.5 and the higher density of nerve fibers, suggests a potential relationship between *ERα36* expression, neural features of lesions, and clinical severity. However, because this study is observational, causal interpretations cannot be made. We propose that ERα36 may represent a candidate molecule involved in pathways relevant to DIE pathophysiology, but mechanistic studies are required to test this hypothesis. Future experimental work will be necessary to clarify whether ERα36 directly influences neural remodeling, lesion progression, or pain generation and to determine its potential value as a diagnostic marker or therapeutic target for endometriosis.

## 5 Conclusion

In conclusion, ERα36 is highly expressed in patients with endometriosis and is significantly associated with DIE. These findings suggest that ERα36 expression correlates with disease severity. However, this observational study does not establish a causal role for ERα36 in the initiation or progression of endometriosis. While ERα36 may represent a potential biomarker of clinical interest, its diagnostic or prognostic value remains to be determined. Further mechanistic studies are required to clarify whether and how ERα36 contributes to the pathogenesis of DIE.

## Data Availability

The original contributions presented in the study are included in the article/**Supplementary Material**. Further inquiries can be directed to the corresponding authors.
