# Supplementary material for: Association between estrogen receptor alpha 36 expression and the risk of deep infiltrating endometriosis
Source: Front Endocrinol (Lausanne). 2026 Mar 11;17:1752870. doi: 10.3389/fendo.2026.1752870 (PMC13034133; doi:10.3389/fendo.2026.1752870)
Supplement: Supplementary file 1 [file DataSheet1.pdf]

PCR gel electrophoresis images

- 25u,NEB Q5 enzyme
- 98℃ 30s
- 98 ℃ 5s
- 65 20s
- 72 ℃ 20s
- 72℃ 2min
- 4 ℃ end

38 cycle (98 ℃ 5s; 65 20s)

CCAAGAATGTTCAACCACAACCT ER1-36-F

GCACGGTTCATTAACATCTTTCTG ER1-36-R

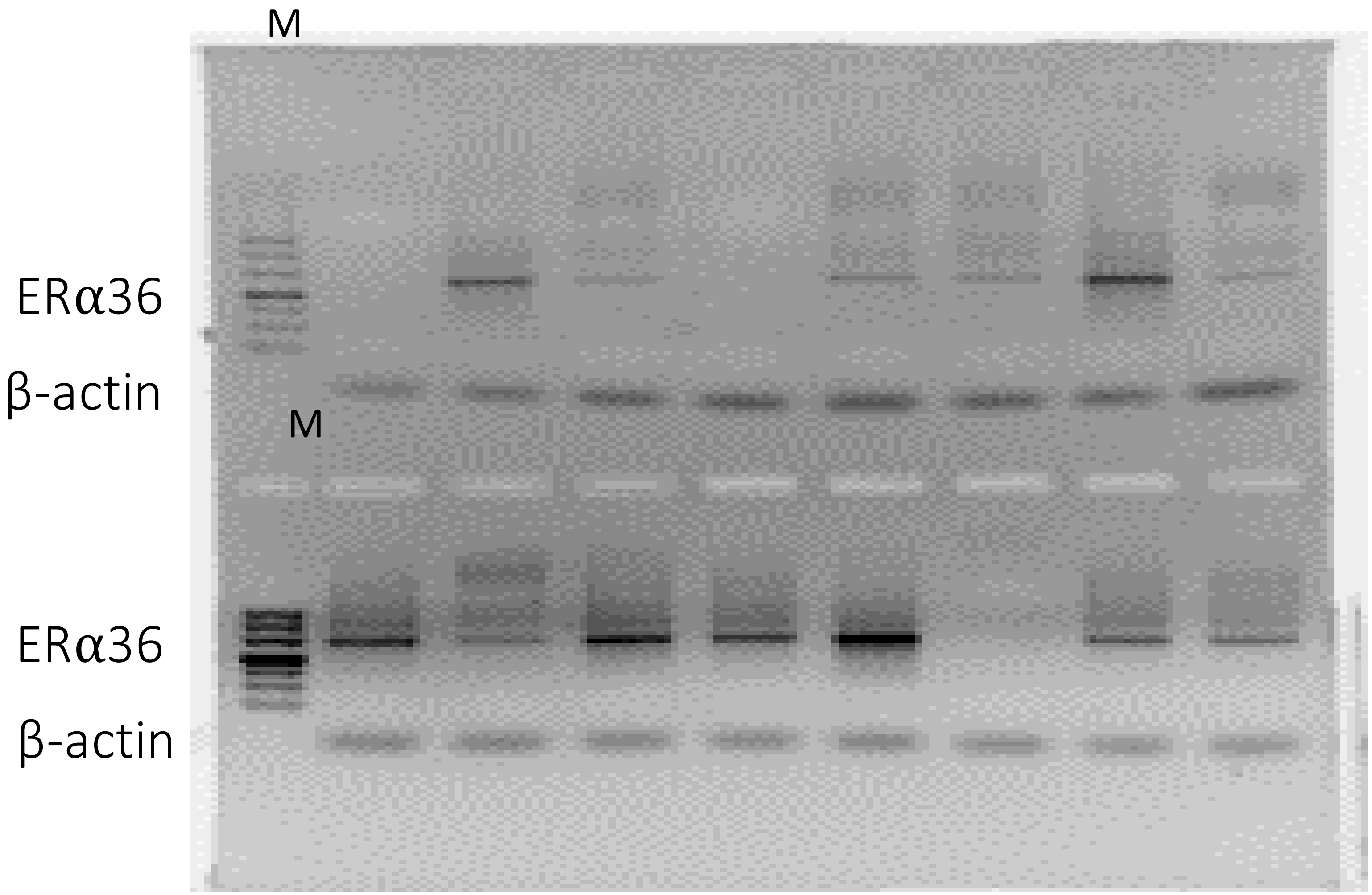
M
ERα36
β-actin
M
ERα36
β-actin

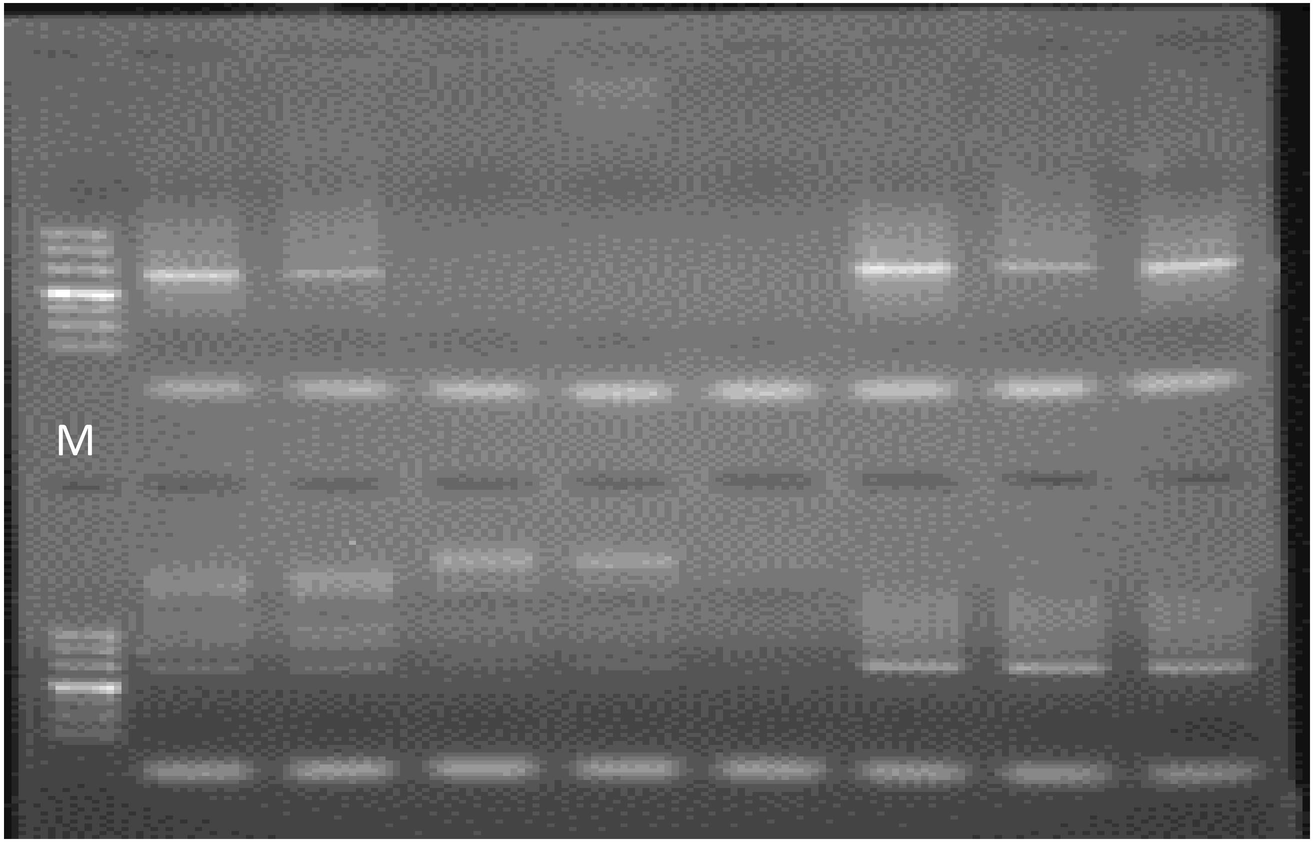

M
ERα36
β-actin
M
ERα36
β-actin

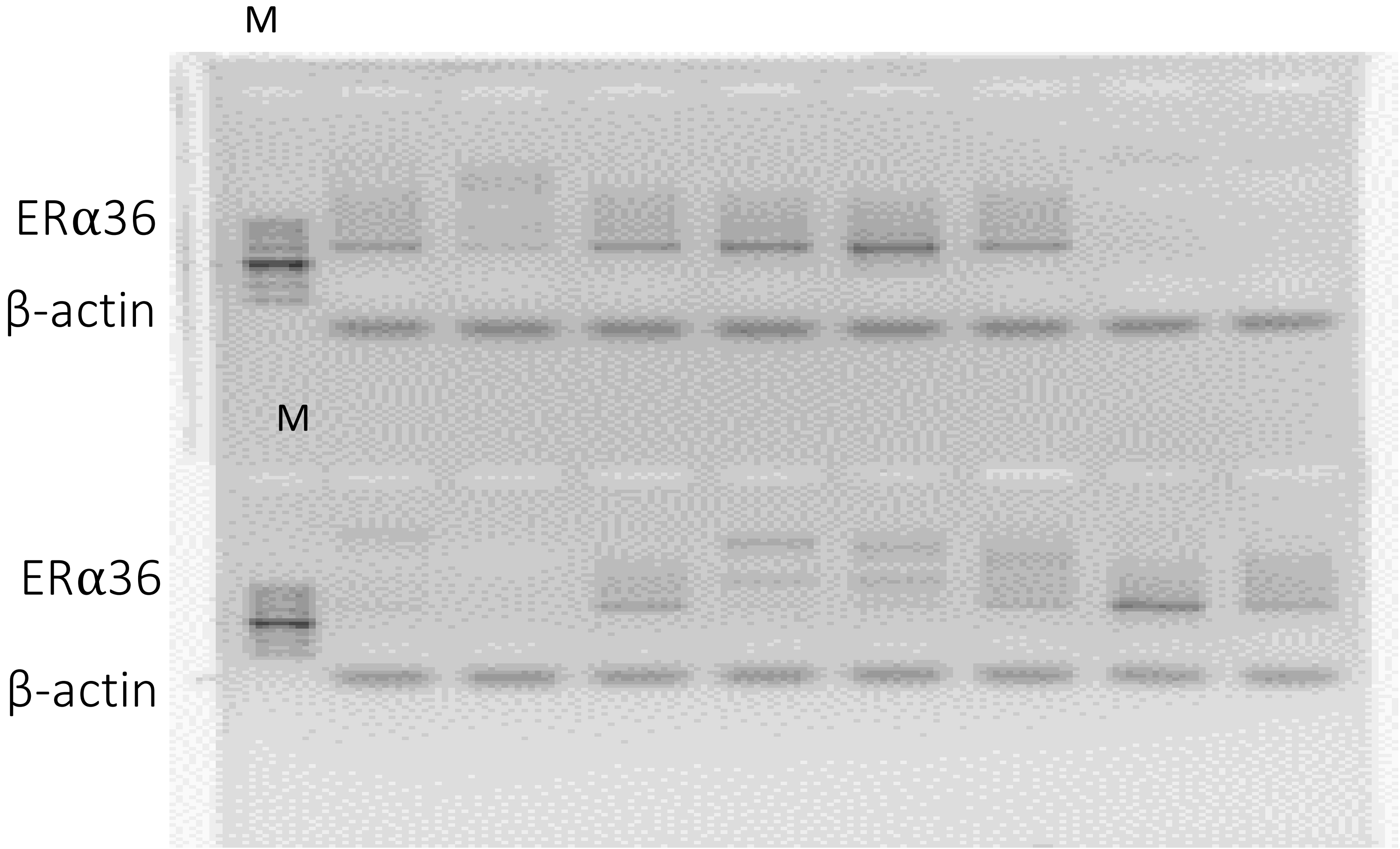
M
ERα36
β-actin
M
ERα36
β-actin

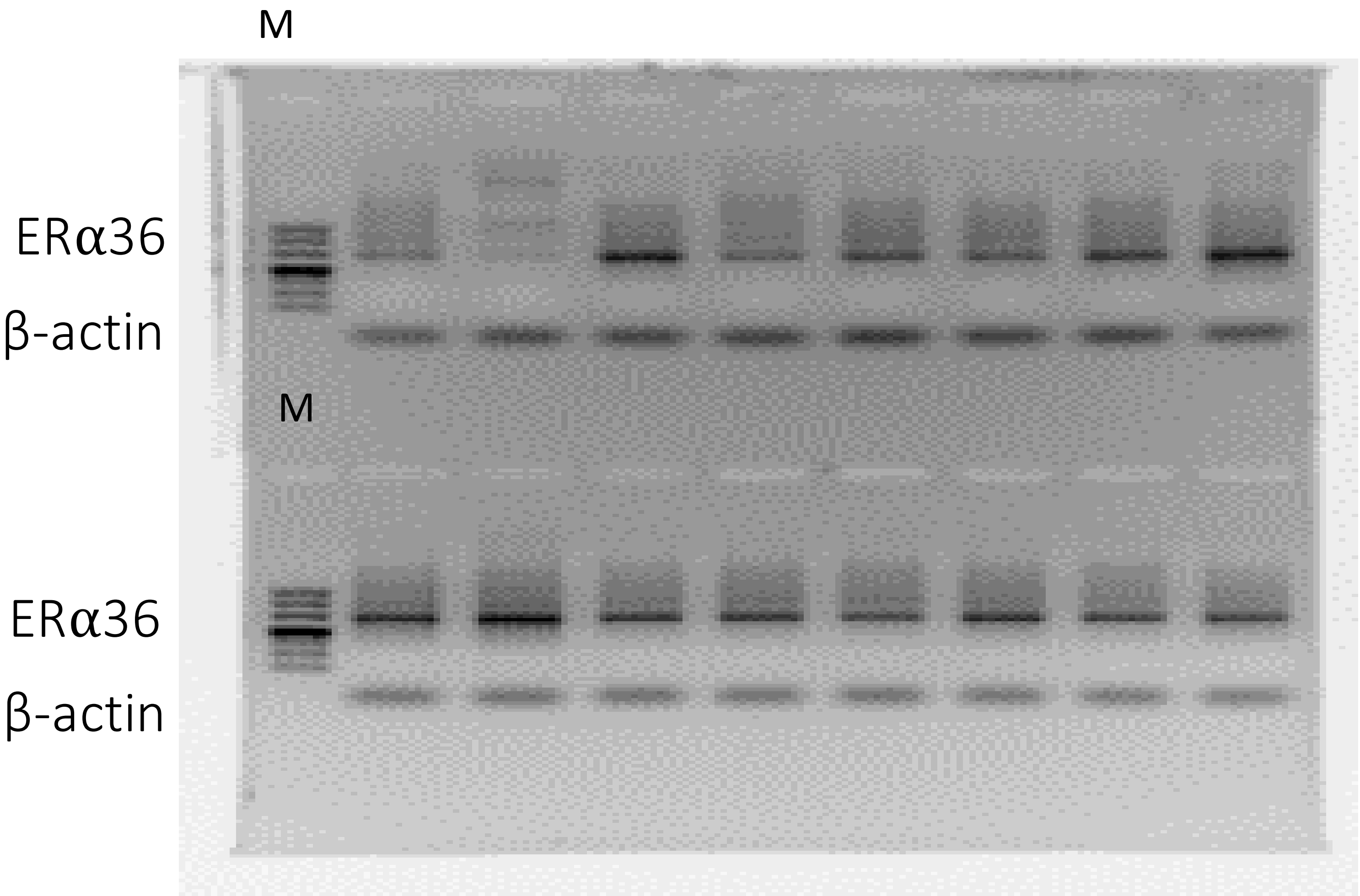

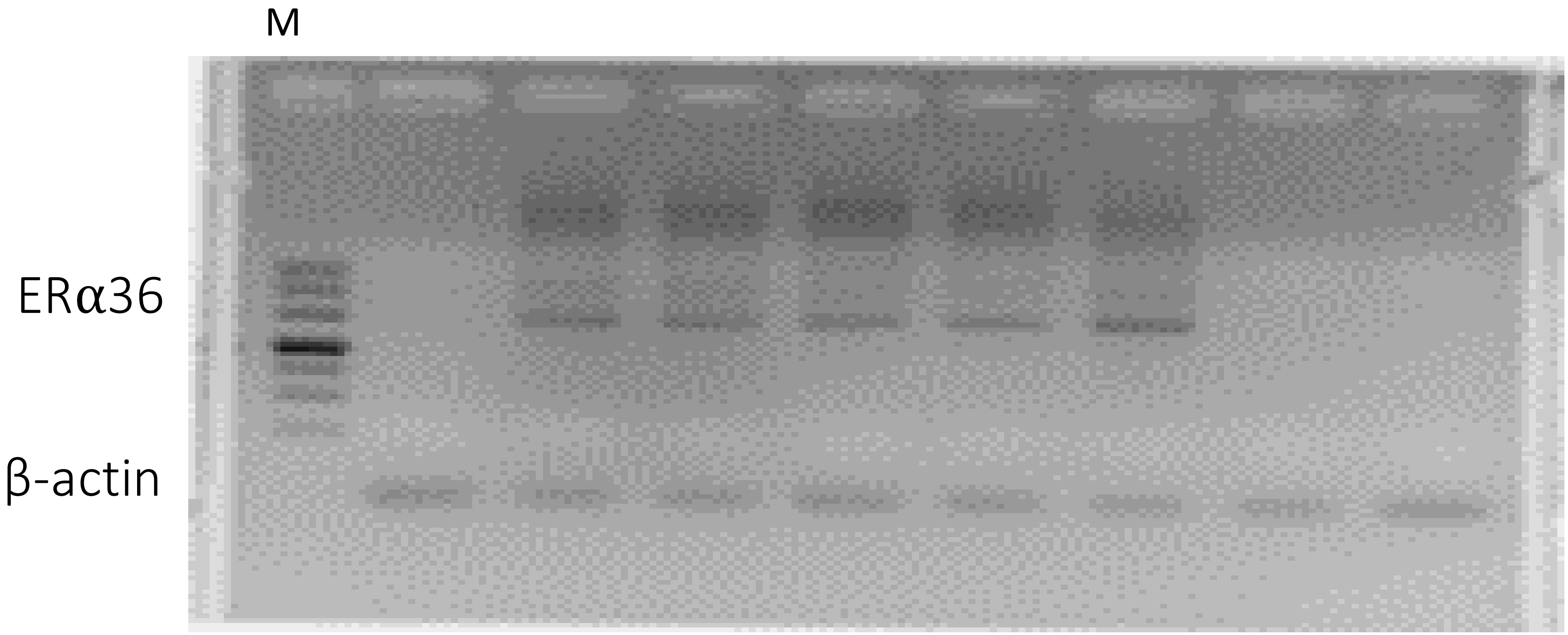
M
ERα36
β-actin

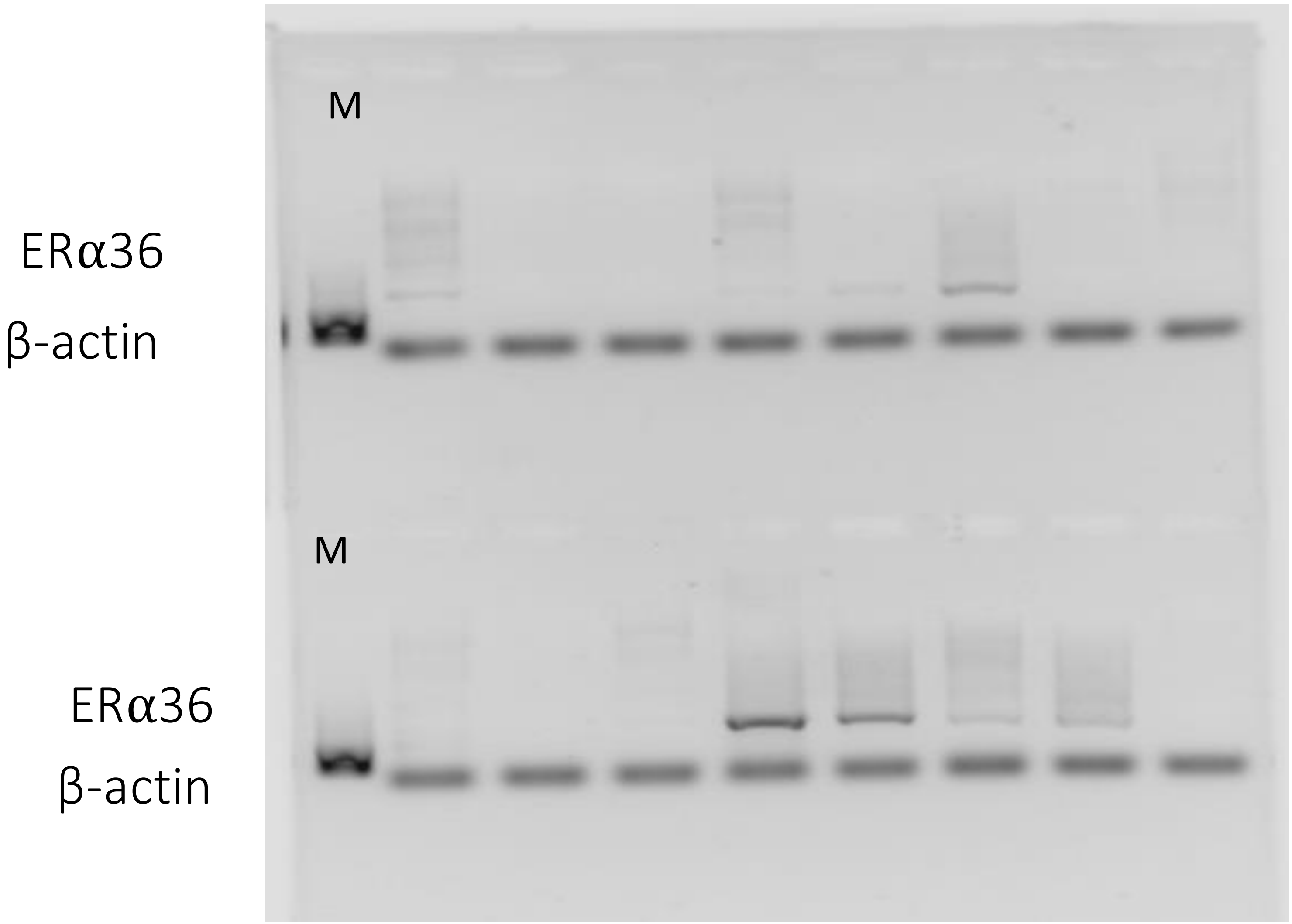
M
ERα36
β-actin
M
ERα36
β-actin

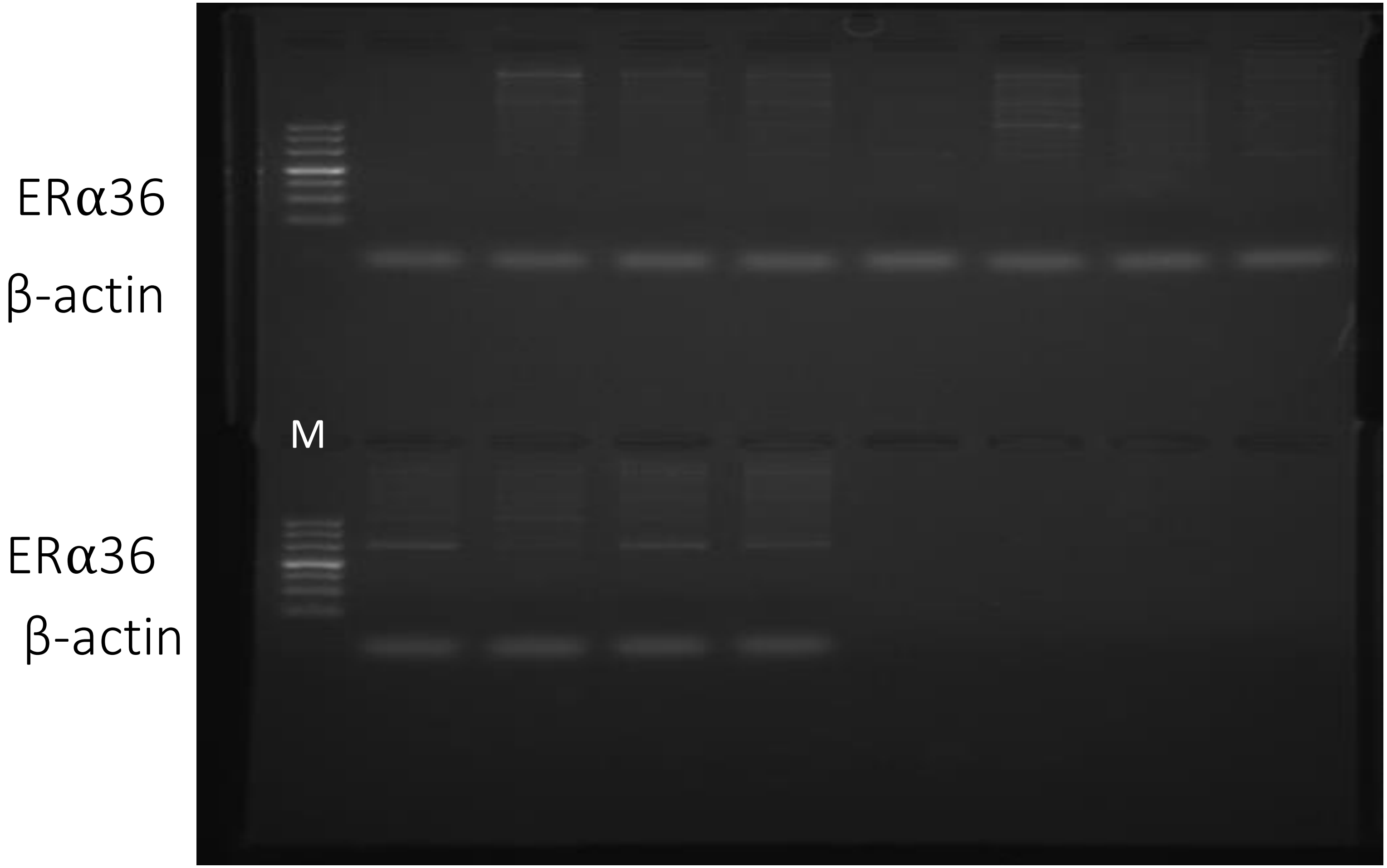
ERα36
β-actin
M
ERα36
β-actin
